# Spontaneous biloma managed with endoscopic retrograde cholangiopancreatography and percutaneous drainage: a case report

**DOI:** 10.1186/1752-1947-5-3

**Published:** 2011-01-06

**Authors:** Gurhan Bas, Ismail Okan, Mustafa Sahin, Ramazan Eryılmaz, Arda Isık

**Affiliations:** 1 Department of Surgery, Vakif Gureba Training and Research Hospital, Istanbul; 2 Department of Surgery, Antalya Training and Research Hospital, Antalya, Istanbul

## Abstract

**Introduction:**

Spontaneous biloma formation is a very rare condition, which mandates immediate treatment.

**Case presentation:**

An 80-year-old Caucasian man was referred to our department with a diagnosis of intra-abdominal collection located in his right upper quadrant. Further radiological examination demonstrated multiple calculi in his gallbladder and common bile duct. Our patient underwent endoscopic retrograde cholangiopancreatography and the stones in the common bile duct were extracted. Percutaneous drainage of the abdominal collection revealed a spontaneous biloma formation. Continuous drainage of bile persisted for one week, so endoscopic retrograde cholangiopancreatography was repeated and a 10Fr stent was placed; subsequently the biliary leak ceased and our patient was discharged. A control abdominal computed tomography did not show any residual fluid collection.

**Conclusion:**

Spontaneous biloma formation is a very rare incidence; awareness is necessary for prompt recognition and treatment.

## Introduction

A biloma is defined as an encapsulated collection of bile outside the biliary tree [1]. It is mainly caused by iatrogenic injury (surgery, percutaneous trans-hepatic interventions) or abdominal trauma [1,2]. Spontaneous rupture of the biliary tree is a very rare condition [3]. We report here the case of a patient with spontaneous biloma formation developed secondary to cholecysto-choledocholithiasis, and managed with percutaneous drainage and endoscopic biliary decompression.

## Case report

**Figure 1:**
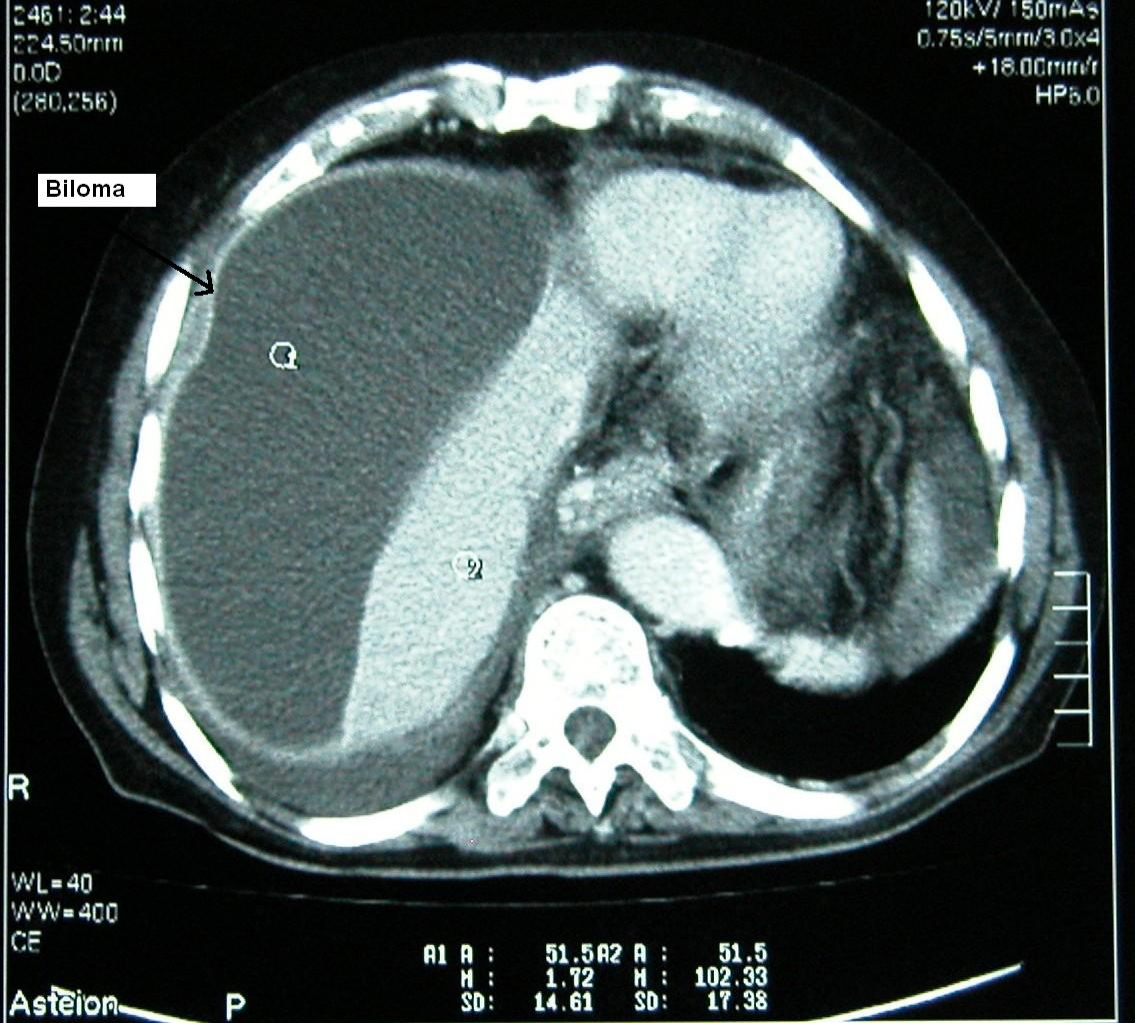
**Initial abdominal CT demonstrating a large right hepatic subcapsular collection**.

**Figure 2:**
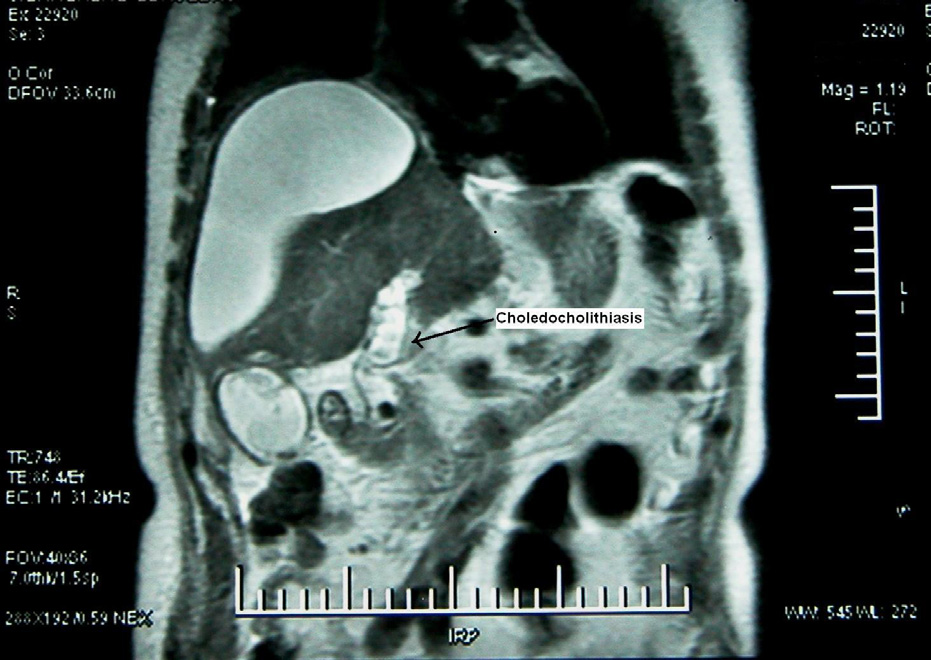
**Abdominal MRI showing CBD stones**.

**Figure 3:**
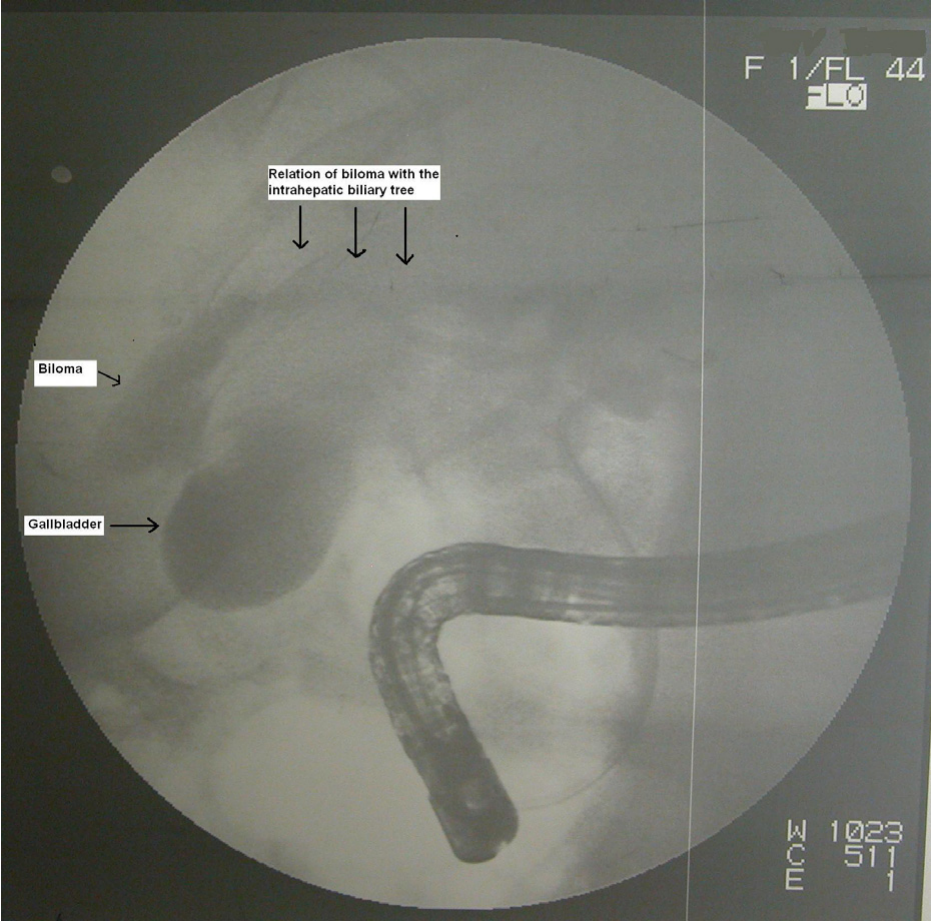
**The ERCP findings reveal relation of the biloma with the intrahepatic biliary tree**.

**Figure 4:**
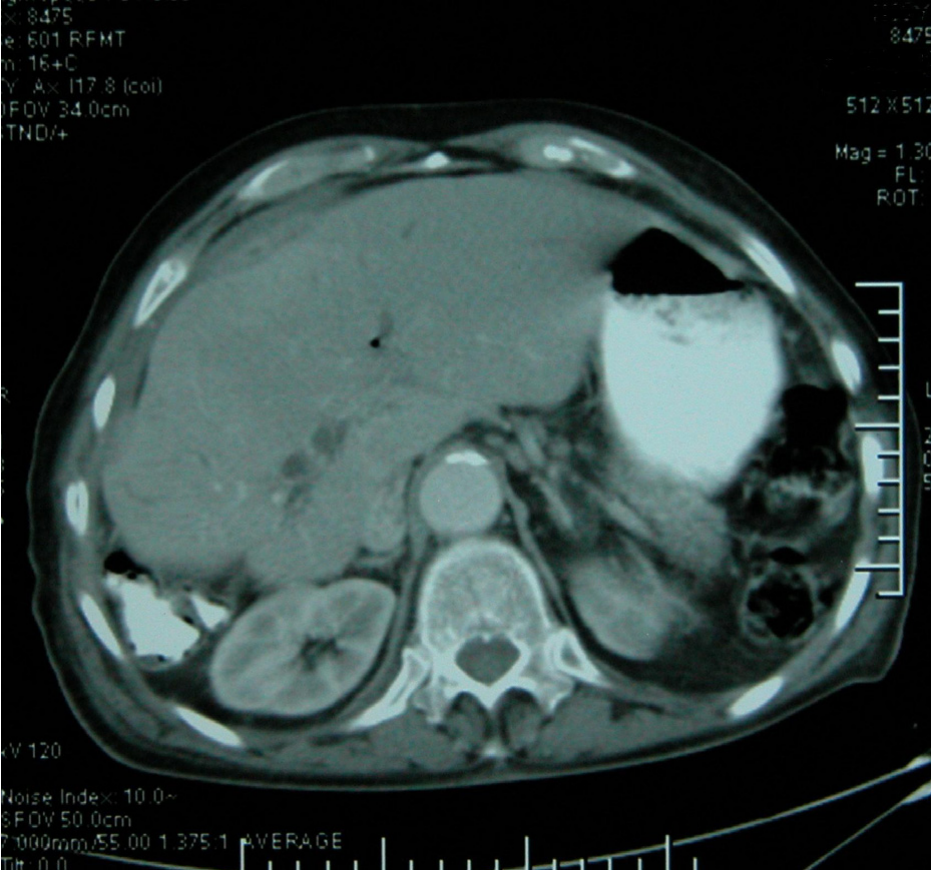
**Abdominal CT showing complete resolution of the biloma after management**.

An 80-year-old Caucasian man was referred to our department with the diagnosis of right upper abdominal encapsulated fluid collection. Two weeks before, he was admitted to the emergency room in a state hospital with abdominal pain and nausea. Subsequent analysis, including abdominal ultrasonography (US) and computed tomography (CT), showed a large fluid collection in his right upper abdominal cavity, and gallbladder stones. He had no past history of abdominal surgery or trauma. On admission, his vital signs and physical examination were normal, except asymmetry and slight tenderness in his right upper quadrant with a palpable mass. Complete blood count and blood biochemistry results were evaluated. Abnormal laboratory findings included (normal range in parenthesis): albumin, 2.3 g/dL (3.5-5.0 g/dL); erythrocyte sedimentation rate (ESR), 82 mm/h; C-reactive protein (CRP), 5.2 mg/dL (0.00-0.80 mg/dL); and calcium levels, 7.7 mg/dL (8.6-1.2 mg/dL). His viral hepatitis marker tests were all negative. A repeat CT revealed a large right hepatic subcapsular collection with a size of 18.9 cm (Figure 1). Abdominal magnetic resonance imaging (MRI) demonstrated multiple common bile duct (CBD) stones with an enlarged biliary tree, and a large subcapsular fluid collection extending around the lower margin of his right hepatic lobe (Figure 2) without any direct communication with the biliary system. Nine days after our patient's admission, endoscopic retrograde cholangiopancreatography (ERCP) and endoscopic sphincterotomy with stone extraction were performed. Two days later, a percutaneous drainage of fluid under US guidance was performed and 800 ml of bile-stained fluid was aspirated. Drain fluid revealed a total bilirubin level of 22.3 mg/dL and a direct bilirubin level of 18.9 mg/dL. Direct microbiological examination with gram staining showed a Gram-negative bacillus. Since a residual collection was detected with US after one week, an 8Fr pigtail catheter was introduced percutaneously. However, daily 50-100 ml drainage continued over seven days, and so a repeat ERCP was performed. It showed extravasation of contrast material from a distal biliary radicle in his right hepatic lobe and communication with the biloma (Figure 3). After the insertion of a 10Fr stent to his CBD, the drainage decreased dramatically and ceased. The percutaneous catheter was removed after five days and our patient was discharged two days later. The 10Fr stent at his CBD was removed two months after his discharge. Control CT scans taken two months (Figure 4) and one year after discharge were normal.

## Discussion

Biloma formation is encountered mainly after surgical or interventional procedures and trauma involving the biliary system [2]. However, there are few reported cases of spontaneous biloma in the literature. The most frequent cause of spontaneous biloma is choledocholithiasis [4,5]. Less commonly reported causes include biliary tree malignancy, acute cholecystitis, hepatic infarction and abscess, obstructive jaundice and tuberculosis [3-5]. Although the pathophysiology of spontaneous biloma remains to be elucidated [5], one suggested contributing factor is an intraductal pressure increase due to obstructive lesions or infarctions on any part of the biliary tree [4]. Bilomas are generally localized in the right upper quadrant of the abdomen, neighboring the right hepatic lobe [4]. The clinical presentation of biloma varies greatly from nonspecific abdominal pain to biliary sepsis [6]. Encapsulation of bile within the omentum and mesentery [2] prevents generalized peritonitis in most cases. Abdominal US is the first modality to evaluate the nature of a biloma and the underlying pathology. However, an abdominal CT can define the disease, the cause and the relations with the adjacent structures more accurately [3]. Differential diagnosis should include hematoma, seroma, liver abscess, cysts, pseudocysts, and lymphocele [5]. Percutaneous aspiration under radiologic guidance can also aid in diagnosis and treatment. Biochemical and microbiological analysis of the fluid helps differentiation from pyogenic abscesses or other causes [7]. An MRI may be of value to evaluate the etiology since it can be used safely for the pathologies of the biliary system [8]. ERCP is also used for diagnostic and therapeutic purposes. Management of the biloma in a patient includes appropriate measures such as intravenous hydration and initiation of antibiotic treatment if sepsis is present. Although some bilomas, especially those that are small in size and asymptomatic, can be followed without intervention [3], most require treatment. Percutaneous [9] and endoscopic modalities provide adequate drainage and may be therapeutic in most cases [6]. These treatments are preferable to surgery as the first step in treatment [4,5,10]. ERCP is indicated particularly in treatment failure, such as persistent bile leakage despite percutaneous catheterization. Surgery always remains an option in emergency and persistent cases. In our patient, the biloma was located in the right upper quadrant and was detected with abdominal US. Because an MRI demonstrated CBD stones, ERCP was preferred for the first modality for diagnosis and treatment. Although it did not show the communication between the biliary tree and the collection and proved biloma, his CBD was cleared from stones. Repeat ERCP with stenting was necessary because the drainage didn't stop. In ERCP, the communication between the biliary tree and biloma was shown clearly, probably due to the decompression of the biloma by percutaneous drainage. The drainage ceased after five days. During our one year follow-up, there has been no recurrence by clinical or radiological means.

## Conclusion

Percutaneous treatment should be considered as the first-line option for patients with symptomatic spontaneous biloma. In cases of persistent bile leaks, ERCP and endoscopic sphincterotomy with or without stent placement should be performed.

## Consent

Written informed consent was obtained from the patient for publication of this case report and any accompanying images. A copy of the written consent is available for review by the Editor-in-Chief of this journal.

## Competing interests

The authors declare that they have no competing interests.

## Authors' contributions

GB, IO, MS and RE analyzed and interpreted the patient data. AI was a major contributor in writing the manuscript. All authors read and approved the final manuscript.
